# Exploitation of Ultrasound Technique for Enhancement of Microbial Metabolites Production

**DOI:** 10.3390/molecules25225473

**Published:** 2020-11-23

**Authors:** Asma Behzadnia, Marzieh Moosavi-Nasab, Shikha Ojha, Brijesh K. Tiwari

**Affiliations:** 1 Department of Food Science and Technology, School of Agriculture, Shiraz University, 7144165186 Shiraz, Iran; 2 Seafood Processing Research Group, School of Agriculture, Shiraz University, 7144165186 Shiraz, Iran; 3 Department of Horticultural Engineering, Leibniz Institute for Agricultural Engineering and Bioeconomy, 14469 Potsdam, Germany; sojha@atb-potsdam.de; 4 Food Chemistry and Technology, Teagasc Food Research Centre, 53.38066 Dublin, Ireland; brijesh.tiwari@teagasc.ie

**Keywords:** ultrasound, microbial metabolites, fermentation, downstream processing

## Abstract

Microbial metabolites have significant impacts on our lives from providing valuable compounds for nutrition to agriculture and healthcare. Ever-growing demand for these natural compounds has led to the need for smart and efficient production techniques. Ultrasound is a multi-applicable technology widely exploited in a range of industries such as chemical, medical, biotechnological, pharmaceutical, and food processes. Depending on the type of ultrasound employed, it can be used to either monitor or drive fermentation processes. Ultrasonication can improve bioproduct productivity via intensifying the performance of living organisms. Controlled ultrasonication can influence the metabolites’ biosynthesis efficiency and growth rates by improvement of cell permeability as well as mass transfer and nutrient uptake rates through cell membranes. This review contains a summarized description about suitable microbial metabolites and the applications of ultrasound technique for enhancement of the production of these metabolites as well as the associated downstream processing.

## 1. Introduction

An extensive range of metabolites produced by various wild-type/engineered microorganisms impacts most life forms and has found ubiquitous roles in our environment [1,2]. These microbial metabolites are widely used in food, pharmaceutics, biofuel, detergents, and pesticide industries [1,3]. Microbial metabolites including amino acids, organic acids, antimicrobial agents, vitamins, rare sugars, and sugar alcohols can be considered as potent alternatives for their chemical counterparts [3]. Different microorganisms can synthesize similar metabolites, for instance, amino acids are produced by *Corynebacterium* [4], *Brevibacterium* [5], and *Escherichia coli* [6]; vitamins are provided by *Propionibacterium* [7] and *Pseudomonas* [8]; organic acids are synthesized by *Aspergillus* [9], *Lactobacillus* [10], *Rhizopus* [11]; suitable enzymes are made by *Aspergillus* and *Bacillus* [12]; antibiotics are prepared by *Streptomyces* [13] and *Penicillium* [14]; and biosurfactants are widely formed by *Pseudomonas* [15], *Bacillus* [16], and *Lactobacillus* [17]. The total global market for microbes and microbial products is expected to reach $250.3 billion by the end of 2023, this market is projected to grow at a compound annual growth rate of approximately 8.7% from 2017 to 2023 (Microbial Products 2020). Considering the huge market demand for these bioproducts, effective production and processing technologies are required [18].

Over the centuries, fermentation techniques have been established and modified for the biosynthesis of various fermentative metabolites. Furthermore, consumer demand is rising for health-care supportive products including probiotic fermented foods. The enhanced demand for such bioproducts has brought competition to the market with a drive to make new products as well as to develop new production processes [19]. The genetic structure of microorganisms and the conditions of the fermentation media influence metabolite/biochemical production. However, there are also an array of process conditions that can be controlled during production to improve the yield of biosynthesis [20]. The physiological actions of a typical microorganism depend on pH, temperature, aeration, and agitation conditions along with the types and concentration of the available nutrients [20]. Genetic manipulation approaches for the enhancement of microbial metabolite production include mutation and recombinant DNA technology. These techniques offer: (1) overexpression of the associated genes involved in the production of the metabolites of interest; (2) knockout of the reactions that degrade the target metabolites; (3) overproduction of the coenzymes (i.e., ATP, NADH and NADPH) that play significant roles in the formation efficiency of the end products; and (4) proficient transport of metabolites outside the cells, which results in the prevention of intracellular accumulation and subsequently any growth-inhibitory effects on the cells [1]. Nevertheless, researchers have been continuously seeking for alternative methods to improve and control productivity. Novel technologies have been tested at various stages of production and have shown beneficial impacts on overall productivities. Application of ultrasonication to control or stimulate living organisms is a relatively new approach [21,22]. Ultrasound is a versatile technique that can be used for process monitoring as well as process intensification in many areas including food, fine chemical, medical, biotechnological, and pharmaceutical industries [19]. Depending on the applied intensity of ultrasound, repairable or unrepairable damages occur in the microbial cells and their surroundings [23,24]. Although the lethal effect of ultrasound on microorganisms has been demonstrated for about a century, the potential of ultrasonication (applying appropriate intensities) to intensify or control their bioactivity was not known until recently [21,22]. Therefore, due to the ability of mixing improvement and mass transfer, especially in biphasic systems, ultrasound is identified as a very useful tool in enhancing the reaction rates in a variety of reacting systems. Ultrasound can operate at mild temperature and pressure conditions and is able to reduce the processing cost and enzyme loading, improve the hydrolysis yield, and mitigate the severity of the pretreatment steps [21,25]. This review paper outlines the application of ultrasound technology in fermentation and downstream process for the improvement of microbial metabolite production and recovery. Various potential applications of ultrasound technology are also discussed.

## 2. Ultrasound Technology

**Figure 1:**
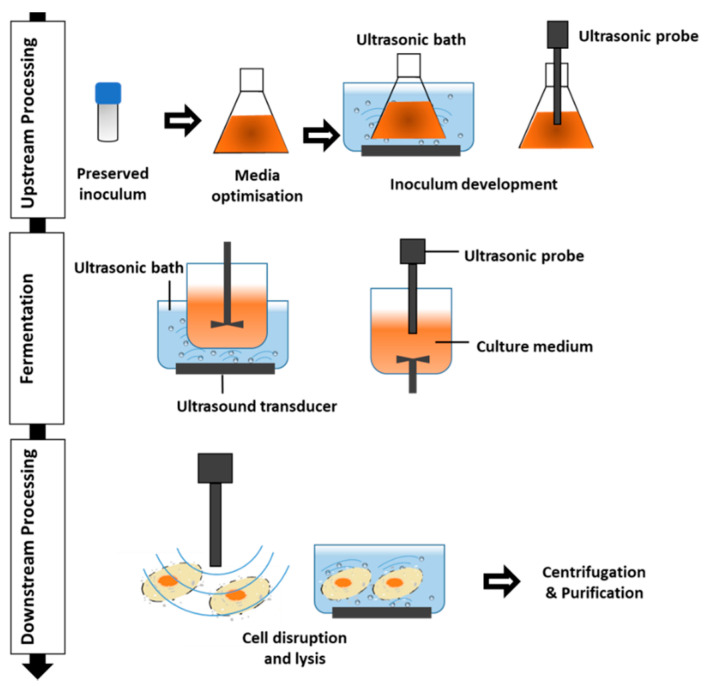
Application of ultrasound in different stages of microbial metabolite processing.

Ultrasound belongs to inaudible sounds to the human ear [26]. The range of ultrasound is divided into: (1) power ultrasound (20–100 kHz); (2) high-frequency ultrasound effectively used for sonochemistry (20 kHz–2 MHz); and (3) diagnostic ultrasound above 1 MHz frequency applied in medical and industrial imaging [21,27,28]. Furthermore, from a practical point of view, ultrasound is generally used over two ranges: low intensity (high frequency 100 kHz to 1 MHz and low power of less than 1 W/cm^2^) as a non-destructive technique to monitor concentration, composition, structure, physical and molecular features; and high intensity (low frequency 20 to 100 kHz and high power of 10–1000 W/cm^2^) in sonochemical processes [21,26,29]. In general, ultrasound is a physical technology with different biological impacts (from destructive to beneficial) depending on the employed intensity [30]. The main effects of ultrasound including chemical and physical changes in the liquid medium are produced via the cavitation phenomenon [31,32,33,34]. It has been demonstrated that ultrasonication stimulates cell permeability, contributing to the release of cellular content from cells. As can be seen in Figure 1, ultrasound has been employed in different stages (using a probe and bath ultrasound systems), stimulating cell permeability contributing to enhancing or releasing cellular metabolites and/or cells. Recognition of the potential application of ultrasound in biotechnology is much more recent, and Sinisterra in 1992 [35] gave an overview on the application of ultrasound in biotechnology and bioprocess for the first time, while the killing effect of ultrasound on living cells has been identified for about a century [21,36,37]. It has been suggested that low intensity ultrasound leads to mass transfer happening through the boundary layer, cellular membrane, and in the cytosol [35]. It has been observed that sonication treatment in bioreactors can increase mass transfer and reaction rates through reducing the boundary layer thickness around the cells located near the bubbles [26]. Furthermore, ultrasonic-induced mass transfer of the reagents to the active site of enzymes and altering their structures can modify the enzymatic activity [35,36]. On the other hand, high intensity ultrasound can cause cell membrane disruption, leading to injury of vital macromolecules (i.e., enzymes, proteins, peptide chains), and subsequently the cells’ inactivation [26]. Thus, mild ultrasonication stimulates biological functions such as enzymatic and microbial bioconversions as well as cellular biosynthesis [30].

The antimicrobial efficacy of high-intensity ultrasound on microorganisms has been demonstrated. However, researchers have also started to investigate low-intensity ultrasound at sub-lethal levels to control and increase cell activities [21]. Low-intensity ultrasound (<2 W/cm^2^ and 70 kHz frequency) was reported to increase the growth rates of *Staphylococcus epidermidis*, *P. aeruginosa*, and *E. coli* cells attached to surfaces, while high-intensity ultrasound (>2 W/cm^2^ and <100 kHz frequency) eliminated cells on the surfaces [38]. Joyce et al. (2011) indicated substantial effects of low-frequency ultrasound (20 and 40 kHz) in dead *E. coli* and *Klebsiella pneumonia* cells, while high-frequency ultrasound (580 kHz) showed deaggregation of bacterial cells more than cell ruptures [39].

## 3. Microbial Metabolites

Microorganisms synthesize primary and secondary metabolites during their different phases of growth. Primary metabolites include amino acids, organic acids, nucleotides, polysaccharides, fatty acids, alcohols, and enzymes [2]. They are essential for the normal growth of microorganisms and are produced in their logarithmic phase of growth (trophophase) [1]. Industrial production of microbial amino acids was started in the 1950s when Kinoshita discovered Corynebacterium glutamicum as a superior amino acid producer. Until this time, amino acids were produced using chemical and extraction methods [40]. After the logarithmic growth phase, microorganisms enter the stationary phase due to depletion in their required nutrients. During this phase, some organisms synthesize suitable secondary metabolites. These metabolites are low molecular compounds associated with secondary cell metabolism (idiophase) independent of its primary one [2]. Secondary metabolites include alkaloids, antibiotics, toxins, pigments, enzyme inhibitors, and antiparasitic agents [2,20]. Inspired by the discovery of antibiotic penicillin by Fleming in 1929, scientists have investigated the therapeutic role of microbial products for combating infections. A large number of microorganisms have been found to possess pharmaceutical capabilities, antimicrobial activities, and other functional properties with a large array of products [2,41]. Table 1 accordingly lists the key primary and secondary metabolites produced by different microorganisms and their functions.

Fermentative production of microbial metabolites meets substantial advantages rather than chemically synthesized counterparts. The fermentation processes can be performed under controlled conditions (nutrient factors and growth conditions) and renewable substrates, leading to the cost-effective and large amounts of product. Moreover, fermentation approaches require a slight amount of energy compared to the chemical processes. Thus, microbial metabolites can be produced on an industrial scale through fermentation technologies [41].

Although the fermentation process is like an old processing technique, it would be a highly competitive, innovative, and leading industry to evaluate the potential of novel technologies to improve fermentation processes. Various novel processing technologies including ultrasound have been investigated to increase the productivity and efficiency of biological processes. The lethal effect of ultrasound on living cells has been demonstrated for years, although the stimulating potential of ultrasound on their activity is much more recent [26,42]. Hereon, utilization of ultrasound at the sub-lethal level that is capable of improving and controlling microorganism activity is one of the most interesting new aspects in ultrasound investigations [35]. Therefore, ultrasound technology has attracted a great interest in terms of a variety potential in biotechnology processes such as the enhancing transportation of oxygen and nutrients into the cell and toxics and by-products out of the cell [21,43].

### 3.1. Antimicrobial Components

Bateriocins are extracellularly-secreted proteinaceous metabolites synthesized in ribosomes by both Gram-negative and Gram-positive bacteria [56,57]. Bacteriocins have both bactericidal and bacteriostatic properties against a range of pathogenic and spoilage bacteria; however, the effectiveness depends on the nature of bacteria. For instance, nisin produced by *Lactococcus lactis* is not effectively active against Gram-negative bacteria because the outer membrane in Gram-negative bacteria avoids nisin action on the cytoplasmic membrane [56,58].

Lactic acid bacteria (LAB) are the most commonly used bacteriocin producers in the food industry due to their abilities to synthesize a wide range of antimicrobial agents that have broad spectrum preservative effects and have a generally recognized as safe (GRAS) status [46,57,59]. To date, a range of bacteriocins produced by different bacteria has been explored. The most extensively studied bacteriocins that have been accepted to be utilized as food additives are nisin and pediocin, synthesized respectively by *Lactococcus lactis* and *Pediococcus* strains [57,60]. Nisin has been approved by the US Food and Drug Administration (USFDA) and it is being used as a food biopreservative in more than 48 countries [60]. According to the potential of LAB to produce a variety of bateriocins, Abbasiliasi et al. (2017) reviewed the influence of various factors and economical aspects on LAB bacteriocin production. They reported significant effects of the growth medium formulation and culture conditions on cell growth and bacteriocin formation. Production of bacteriocins occurs mainly during the exponential phase; hence, it conforms primary metabolism as cell growth-associated metabolites [56].

### 3.2. Bioethanol

Biofuels have been promoted as environmentally-friendly solutions to fossil fuels. Bioethanol is a fuel replacement that has been used in Europe and the United States since the early 1900s [61]. Although bioethanol is an interesting replacement for gasoline, it is currently more expensive than the conventional fuels at commercial scale. Lignocellulosic biomass is the most widespread natural organic resource that consists of cellulose, hemicellulose, lignin, proteins, and inorganic minerals and can significantly reduce the production costs. Generally, bioethanol production is a multi-step process including the pretreatment of biomass, hydrolysis of cellulose and hemicellulose, and fermentation of hydrolysate to bioethanol. Each step has a remarkable effect on the produced bioethanol [61]. Due to its complexity, biomass needs to be broken down prior to conversion to fermentable carbohydrate. Dissociation and accessibility of lignocellulosic biomass by using chemical, mechanical, and thermal methods have been reported; however, most of these approaches also release undesirable products, and contribute to the loss of some important components. Thus, it is desirable to explore and apply novel technologies offering more favorable economic and environmental aspects [62].

### 3.3. Biosurfactants

Biosurfactants are surface active agents produced by various microorganisms such as bacteria, fungi, and yeasts as by-products of the biotransformation of organic substrates [63,64]. These components consist of a hydrophilic head comprising saccharides, acids, peptide anions, or cations and a hydrophobic tail comprising saturated or unsaturated hydrocarbons or fatty acids [65]. Their structural diversity includes lipopeptides/lipoproteins, glycolipids, proteins and polysaccharides, and lipopolysaccharides, of which glycolipids are well-known biosurfactants [66]. Rhamnolipids produced by Pseudomonas aeruginosa are the most studied biosurfactants commercially available as fungicides for agriculture or as emulsifiers for bioremediation activities [65].

Surfactants are ubiquitously employed across the industrial sectors including agriculture [64,67]. They can decrease the surface and interfacial tensions between different interfaces such as oil/water and air/water [67]. Nowadays, most industries use petrochemical-based surfactants. However, these surfactants are non-biodegradable, toxic to most living organisms, and classified as environmental hazards. Consequently, a search for environmentally-friendly alternatives is underway [68,69,70].

Compared to chemical surfactants, biosurfactants have many advantages like lower toxicity, higher biodegradability, and being produced through greener processes and effective activity over wide ranges of pH, temperature, and salinity [63,67,71,72]. Biosurfactants are also used as complexing agents for the recovery of heavy metals, emulsifiers for the accessibility of hydrophobic molecules, wetting and foaming agents, food ingredients, and detergency agents in several industries [67]. Moreover, these compounds have several therapeutical and biomedical properties including possessing antiviral, antibacterial, and antifungal properties along with anti-adhesive actions against various pathogenic microorganisms [71]. Madhu et al. (2014) observed anti-adhesive effects and antimicrobial activities for a glycoprotein biosurfactant produced by *L. plantarum* against some food-borne pathogens including *E. coli* ATCC 31705, *E. coli* MTCC 108, *Salmonella typhi*, *Yersinia enterocolitica* MTCC 859, and *Staphylococcus aureus* F 722 [50].

Over the past few years, researchers have discovered several biosurfactant-producing microorganisms in addition to worthy insights on their production, types, and properties [71]. Nevertheless, large-scale production of microbial biosurfactants remains challenging due to low process efficiencies and high production expenses [71].

### 3.4. Other Microbial Metabolites

Enzymes are large biological catalysts that facilitate all important chemical interconversions required to sustain life by accelerating the rate and specificity of metabolic reactions. Enzymes catalyze almost all chemical conversions, lowering the activation energy of the reaction range from the digestion of food to the synthesis of DNA [73,74].

Several microorganisms including bacteria, actinomycetes, fungi, and yeasts synthesize a variety of enzymes with various structural and commercial applications. Amylase, protease, pectinase, lipase, xylanase, cellulose, and laccase are produced extracellularly, while catalase is intracellularly synthesized by *Saccharomyces cerevisiae* and *Aspergillus niger* [75,76]. Approximately 85% of industrial enzymes are produced by bacteria, fungi, and yeast and the remaining 15% are made by plants and animals. Microbial enzymes consist of several advantages over those produced by animals and plants as follows: (i) more activity and stability; (ii) higher production yield; (iii) easy for modifying the characteristics by protein engineering; and (iv) applying modern techniques like metagenome screening and genome mining for exploring microbial enzymes [76].

Vitamins are essential micronutrients that are not synthesized by mammals, but by microorganisms and plants. These micronutrients are essential to retain an equilibrated metabolism in all living forms [2,77]. At present, vitamins and vitamin-related compounds are increasingly used in the food, feed, pharmaceuticals, cosmetics, and medical-therapeutic agents industries given their vital nutritional and physiological roles [77]. Vitamins are required in trace quantities to retain normal physiological action of the body [2,77]. Some vitamins are industrially produced by chemical and extraction methods; however, such methods are energy-intensive and produce significant waste. Increased awareness by consumers for natural additives has resulted in the replacement of these methods with biotechnological approaches. Currently, biotechnological production of vitamins and vitamin-related compounds are effectively competing with chemical processes [77].

Organic acids are used as ingredients in several industries (food, beverages, pharmaceuticals, textile, detergents, perfumes, plastics and adhesives). Microorganisms have a great potential to commercially provide several organic acids including lactic acid, acetic acid, citric acid, and gluconic acid [2]. Presently, fermentative production of some organic acids is much more widespread than chemical processes [2].

## 4. Application of Ultrasound for Improving Productivity of Microbial Metabolites

Numerous methods have been reported to improve the productivity of fermentative products. The application of ultrasound to control or stimulate living organisms is a relatively new approach [21,26]. Depending on the applied intensity of ultrasound, repairable or unrepairable injures are provided on the microbial cells and their surroundings. Unlike the high intensity ultrasound, low intensity ultrasound is able to accelerate the proliferation of microbial cells, leading to enhancement of the products’ metabolism [23,24,78]. It has frequently been suggested that the effect of ultrasound increases fluid convection and transport of molecules through the boundary layer of the liquid surrounding the cells [26,38,79]. Table 2 lists key examples of ultrasound applications to induce bioprocess productivity.

In general, increased convection through the membranes stemming from ultrasound leads to the enhanced transport of oxygen and nutrients to the cells and also the transfer of waste products out of the cells [38].

**Figure 2:**
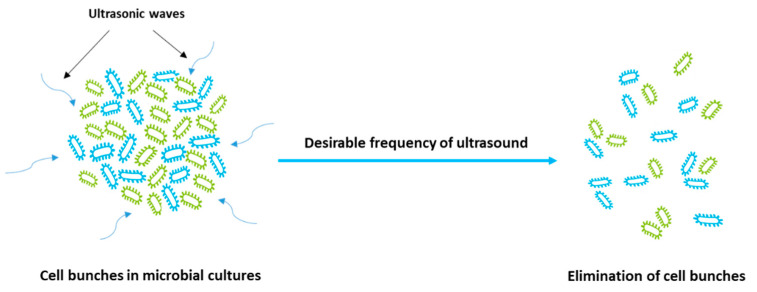
Effect of ultrasonication on the elimination of microbial cell bunches.

The profitable (positive) effects of ultrasound on microbial metabolism are considered to be a combination of several mechanisms including:

(i) Elimination of cell bunches in microbial cultures (see Figure 2) by which enhanced nutrient exploitation by growth medium leads to increased growth cell, biomass concentration, and productivity of microbial metabolites.

(ii) Increasing the cell membrane permeability, resulting in nutrient uptake through the membrane and leading to boost cell growth and proliferation [24]. Behzadnia et al. (2019 & 2020) presented improved penetration of the cell, then stimulation of the transfer action of the cell, thus enhancing the substrate uptake and thereby promoting the growth and generation of cells [85,92]. Sonication of microbial cells was found to increase cell permeability, which induces the transfer action of cells, leading to the rise of substrate uptake, followed by improved growth and cell regeneration, and subsequently enhanced biomass concentration [85,93]. The ultrasonic waves break the ingredients into smaller compounds, thereby the nutrient and oxygen uptake rate increase through the cell membrane. Moreover, pulsation of microbubbles results in the reduction of solid–liquid and gas–liquid mass transfer resistance through the cells [26].

(iii) Improving the condition of the culture medium, making it a desirable environment for the growth and proliferation of cells [24]. For instance, Lanchun et al. (2003) found pH and foam induced by ultrasound as key factors playing crucial roles in the growth of *Saccharomyces cerevisiaes* by modifying the solubility and accumulation of carbon dioxide as well as improving the exchanges between gas and liquid phases [94].

(iv) Speeding up the proliferation of microbial cells by influencing the involved cellular constituents and traits [24,95].

**Figure 3:**
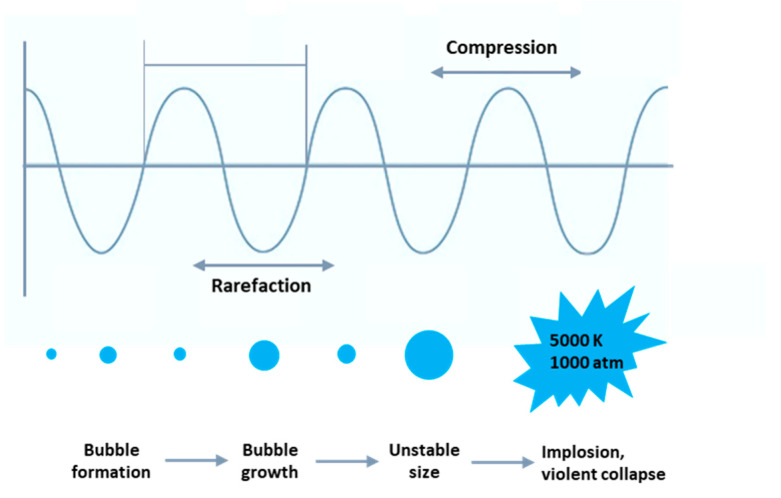
Mechanism of the cavitation phenomenon creation.

Most of the effects of ultrasound result from mechanical and hydrodynamic reactions created by acoustic cavitation and microstreaming [30,96]. Propagation of high-power ultrasound passing through a liquid medium generates alternating compression and rarefaction cycles (see Figure 3). During the rarefaction cycle, small bubbles are produced during sonication. Indeed, the interactions between the ultrasonic waves and liquid and gas/vapor lead to diffusion of gas/vapor into the oscillating bubbles and then quick growth and subsequently in the next compression phase, the bubbles implode violently and collapse [97]. During implosion, shock waves (with high energy density), extremely high temperatures (up to 5000 K), and high pressures (almost 1000 atm) are created in very short events at a localized spot of the medium, which are able to induce chemical and mechanical effects [26,29]. The localized events (high temperatures and high pressures) change extremely rapidly at >110 °C s^−1^. The formation, expansion, and implosion of bubbles cause the cavitation phenomenon [26,36]. Acoustic frequency is an important factor in the formation of cavitation bubbles. Hence, low-frequency ultrasound (for example 20 kHz) induces larger cavitation bubbles than the high-frequency one (for example 580 kHz), giving larger acoustic cycles and longer time durations required for the formation of cavitation bubbles [39,98]. Consequently, lower frequencies such as 20 kHz causes greater mechanical and thermal effects to bacterial cells than the 580 kHz frequency. It has also been reported that the medium composition, viscosity, sound transfer, and power distribution within the reaction solution and also the shape of the bacteria influences the sensitivity of cells to ultrasound irradiation. Larger bacteria have larger surface area exposure to sonication, resulting in a higher sensitivity than the smaller ones. Cocci-shaped bacteria have been reported to be more resistant to ultrasound than bacilli-shaped ones [39]. As mentioned before, lignocellulose biomass is the most abundant natural source for biofuel production; nevertheless, the main obstacle in its use is the recalcitrance of lignocellulose, which leads to low thermal conductivity and the necessity of harsh conditions and solvents [99]. The alteration of the chemical and physical characteristics of lignocellulosic substrate and enhanced saccharification of cellulose by means of substrate pretreatment using 20 kHz high-power ultrasound have been represented [82].

In lignocellulose biomass processing, the ultrasound technique is able to increase catalytic activity and accelerate reaction rate by using the facilitation of pretreatment, fractionation, chemical reactions, and intensification of heat and mass transfer. These actions cause a higher efficiency of biofuel production [99]. It was found that low-intensity ultrasound (11.8 Wcm^−2^) is an effective and sustainable process for ethanol production from lactose by the yeast *Kluyveromyces marxianus* (ATCC 46537). This was utilized during batch fermentation with 10%, 20%, and 40% duty cycles using a sonotrode tip. Sonication at 10% and 20% duty cycles significantly affected the cell growth and biomass concentration, while a 40% duty cycle had a reduced effect on both cell growth and biomass production. The observed impacts can be attributed to the low rate of lactose consumption resulting in an earlier end of the exponential growth phase. The most effective duty cycle (20%) produced an ethanol concentration of 5.20 ± 0.68 g L^−1^, nearly a 3.5-fold increase compared to the control sample. The improved gas–liquid mass transfer conditions were proposed to lead to greater biomass concentrations and bioethanol production. In this study, they concluded that the removal of carbon dioxide resulted from improved dissolved oxygen mass transfer, since high concentrations of carbon dioxide had inhibitory effects on *Saccharomyces cerevisiae* and *K. marxianus* [83]. Maddikeri et al. (2015) used ultrasound irradiation (40 kHz at 600 W power and 20% duty cycle) to enhance sophorolipid biosurfactant production employing *Starmerella bombicola*. It was found that the biomass and sophorolipid concentrations significantly increased with the application of ultrasound during the exponential growth phase. The findings suggest that the alternation of cell permeation and decreased mass transfer resistance through the cell wall result in an increased substrate uptake and gas-liquid transportation [63]. It has been found that power ultrasound affects lactose hydrolysis, which is linked to a release of intracellular enzymes to the fermentation medium. Lactose hydrolysis occurs inside the cell using intracellular β-galactosidase. Exposure to ultrasound decreases the mass transfer resistance through the cell membrane, with release of β-galactosidase to the fermentation medium and an increase in the lactose hydrolysis rate [100,101], resulting in a decrease in the fermentation time by 30 min compared to the control and subsequently may improve in cost reduction in the biotechnology industry [100,102]. Although it is difficult to realize ultrasound transfer through the cell, we can achieve some information by measuring the change in biochemical properties. Chuanyun et al. (2003) obtained significant findings in riboflavin production using fungus *Ecemothecium ashbyii* affected by 24 kHz frequency ultrasound. It was concluded that ultrasound treatment affects biomass concentration (from 11.435 to 15.213 g L^−1^), fermentation time (from 90 to 60 h), glucose and nuclear acid content, and also riboflavin production (between 0.2480 and 0.6862 mg L^−1^) at a 24 kHz frequency compared with the control (without ultrasound). Biochemical changes from this study reflected positive effects of ultrasound on cell growth, metabolism, and cell numbers of *E. ashbyii* [103]. Low-energy ultrasound (0.6–0.8 J/cm^3^) has been reported to induce ion flux (Ca^2+^ influx, K^+^ efflux/H^+^ influx) through the membrane of *Panax ginseng* cells and active oxygen species production. In addition, biosynthesis of ginseng saponins and useful secondary metabolites were stimulated under these conditions. The results of this research indicated a stimulation of the defense responses of plant cells using ultrasound [30].

## 5. Ultrasound Technology for Downstream Processing

The application of ultrasound for downstream processes is a promising method compared to the addition of chemicals and enzymes, which can result in product contamination and involves high costs. On the other hand, high temperatures from thermal and microwave processes lead to loss in end product qualities. Hence, ultrasonication offers an attractive technique that is more acceptable and expandable for continuous processing due to the lack of need to add external compounds (enzyme, chemical) and operation at lower temperatures. Long exposure time durations may create significant amounts of free radicals and degradation of components like oil [104]. Recently, the functional properties of algae have attracted attention with potential applications in biofuels, cosmetics, nutraceuticals, and pharmaceuticals. Ultrasound has been found useful for improving the extraction efficiency of beneficial components including lipid and protein from algae [104,105,106]. Ultrasound has been reported for the production of biofuel from *Dunaliella salina* and *Nannochloropsis oculata,* two species of microalagae. Results from confocal microscopy indicate partial cell destruction and increased resistance of *N. oculata* to ultrasound at 20 kHz and declumping (5–40%). Lipid secretion under controlled ultrasound conditions can support biofuel production [105].

The impacts of ultrasound on the extraction of functional components has been reported in many publications and are associated with higher-yield extractions and faster processing [107]. Ultrasound increases mass transfer though the cells and also mixing of the liquid and solid in the medium, providing higher driving forces and turbulence [107,108]. Ultrasound induced stevioside extraction of *Stevia rebaudiana* resulting in improved productivity over conventional soaking by two hundred times and also a lower performance time [107]. It is well-established that microbial cell lysis occurs with ultrasound via extraction of intracellular molecules. This method has been reported for efficient and aseptic extraction of bacterial enzymes from some bacterial genera (Anonymous, 2000).

Smirnou et al. (2017) facilitated downstream processing of high purity schizophyllan from *Schizophyllum commune* using ultrasound in culture broth. Ultraonication decreased the culture broth viscosity, resulting in faster filtration and almost zero product loss during filtration [80]. Online ultrasonic treatment significantly improved gentamicin productivity from *Micromonospora* spp. by 1.7 times. Ultrasound by the secretion of intracellular gentamicin through the cell wall decreased accumulation of intracellular content and consequently increased gentamicin biosynthesis [109].

## 6. Conclusions

Ultrasound technology, operating at either low intensity or high intensity can induce various effects (e.g., stimulation and inactivation) of microorganism metabolism. Although it has been shown that ultrasound can significantly influence growth rate and production efficiency through increased permeability of cells, mass transfer across the cell membrane, nutrient uptake, and waste release, the precise mechanisms involved are not completely understood. It is necessary to understand that an adequate ultrasound condition plays a crucial role in the consideration of the inactivation of microorganisms, enhancement of cell growth, and improvement of downstream efficiency. Despite the high performance costs of ultrasound in wide industrial adoption, these can be easily compensated by increased productivities. Moreover, the use of inexpensive substrates for the fermentation process as well as the lack of additional chemicals and enzymes for downstream processing may balance the costs of scaling up.

## Figures and Tables

**Table 1:** Synthesized microbial metabolites.

Metabolite	Source	Properties	Reference
Nisin	*Lactococcus lactis*	Inhibitory activity against food spoilage and pathogenic bacteria	[42]
Bacteriocin-like inhibitory compounds	*Bifidobacterium*	Active against Gram-positive, Gram-negative bacteria and yeasts Resistant to α-amylase and lipase	[43]
Proteinaceous bacteriocin-like substance	*Enterococcus faecium*	Inhibitory activity against Gram-positive bacteria (*Listeria monocytogenes*, *Staphylococcus aureus* and other enterococci)	[44,45]
Plantaricin	*Lactobacillus plantarum*	preservatives in canned foods	[46]
Reuterin	*Lactobacillus reuteri*	Biopreservative in fermented milk products	[47]
Antimycin-A antibiotic	*Streptomyces olivaceiscleroticus*	Antifungal agent	[48]
Rhamnolipid biosurfactant	*Pseudomonas aeroginosa*	Surface tension reduction Bioremediation activity in the marine environment	[49]
Glycoprotein biosurfactant	*Lactobacillus plantarum*	Emulsification, Antimicrobial, Antiadhesive properties	[50]
Surfactin biosurfactant	*Bacillus subtilis*	Surface tension reduction	[51]
Bioethanol	*Saccharomyces cerevisiae*	Without dangerous element in acceptability as a fuel Less environmental effects	[52]
Cellulase	*Aspergillus niger* & *Trichoderma* sp.	Saccharification	[53]
β-galactosidase	*Kluyveromyces marxianus*	Hydrolysis of lactose in dairy industry	[54]
Bacitracin antibiotic	*Bacillus* sp.	Antibiotic activity against *Micrococcus luteus* and *Staphylococcus aureus*	[55]

**Table 2:** Application of ultrasound technology for the production or activation of various microbial metabolites and functions.

Metabolite/Activity	Processing Conditions	Salient Findings	References
β-1.3(1.6)-Glucan schizophyllan (SPG)	20 kHz, 2000 W, 100% amplitude	High purity SPG having immunomodulatory activity	[80]
Ethanol production	24 kHz, 60% amplitude	Inducing enzymatic hydrolysis of sugar/maximum yield of 90% ethanol	[81]
Bioethanol production	40 kHz, 5 min, 60 °C	Accelerated the starch hydrolysis, degradation of starch granules and release of glucose, Increase the ethanol concentration by 11.15%	[82]
Bioethanol	1.8 Wcm^−2^ 20% duty cycle	Enhanced the extracellular and the intracellular levels of β-galactosidase Ethanol concentration of 5.20 ± 0.68 g L^−1^	[83]
Sludge activity	35 kHz, 0.2 W/cm^2^, 10 min	Enhanced biological removing the chemical oxygen demand (COD)	[84]
Saponins of ginseng cells	≤0.1 W/cm^3^, 38.5 kHz	Increased cross-membrane ion fluxes (Ca^2+^ influx and K^+^ efflux/H^+^ influx) Production of active oxygen species, Stimulation of useful secondary metabolites synthesis	[30]
Sophorolipid biosurfactants	40 kHz, 600 W, 10 min at duty cycle of 20%	Increased sophorolipid production by 193%	[63]
Biosurfactant	25 kHz, 7.4 W, 30 min	Increased biomass and biosurfactant production by 1.3 times	[85]
Rhamnolipid biosurfactant	150 W, 6 min, 42.5% duty cycle	Enhanced the yield of rhamnolipid 1.5 folds	[86]
Fibrinolytic enzyme	25 kHz, 160 W, 20% duty cycle for 5 min	Improving substrate intake and metabolism of microbial cell Increased productivity of fibrinolytic enzyme by 1.82-fold	[87]
Galactooligosaccharide enzyme	30% amplitude, 30 W	high yield of galactooligosaccharide production	[88]
Lactoperoxidase purification	Intermittent 35 kHz and 250 rpm, 25 ± 2 °C	Purification of lactoperoxidase by coupling aqueous two-phase extraction Increasing in flux	[89]
Fermentation profile of *Lactobacillus sakei*	Low power ultrasound (2.99 W) for 5 min	Higher specific growth rate (µ) and shorter lag phase Antimicrobial activity of Cell-free extracts against pathogenic bacteria	[90]
Apple juice fermentation	Pulse duration 0.5 s and 6 s rest period	Increase in biomass growth and glucose consumption	[91]
